# Discovering Link Communities in Complex Networks by an Integer Programming Model and a Genetic Algorithm

**DOI:** 10.1371/journal.pone.0083739

**Published:** 2013-12-30

**Authors:** Zhenping Li, Xiang-Sun Zhang, Rui-Sheng Wang, Hongwei Liu, Shihua Zhang

**Affiliations:** 1 School of Information, Beijing Wuzi University, Beijing, China; 2 National Center for Mathematics and Interdisciplinary Sciences, Academy of Mathematics and Systems Science, Chinese Academy of Science, Beijing, China; 3 Department of Physics, The Pennsylvania State University, University Park, Pennsylvania, United States of America; Wayne State University, United States of America

## Abstract

Identification of communities in complex networks is an important topic and issue in many fields such as sociology, biology, and computer science. Communities are often defined as groups of related nodes or links that correspond to functional subunits in the corresponding complex systems. While most conventional approaches have focused on discovering communities of nodes, some recent studies start partitioning links to find overlapping communities straightforwardly. In this paper, we propose a new quantity function for link community identification in complex networks. Based on this quantity function we formulate the link community partition problem into an integer programming model which allows us to partition a complex network into overlapping communities. We further propose a genetic algorithm for link community detection which can partition a network into overlapping communities without knowing the number of communities. We test our model and algorithm on both artificial networks and real-world networks. The results demonstrate that the model and algorithm are efficient in detecting overlapping community structure in complex networks.

## Introduction

In the past, it has been shown that many interesting systems can be represented as networks composed of nodes and links, such as the Internet, social and friendship networks, food webs, and citation networks [1]–[3]. An important topic of current interest in the area of networks has been the idea of communities and their detection. Detecting communities from a network is a universal problem in many disciplines from sociology, computer science to biology [4]–[6].

Typically there are two kinds of communities which are node communities and link communities respectively. A node community is a dense subgraph induced by a set of nodes, where nodes are densely connected within the subgraph, but sparsely connected with nodes outside of the subgraph. Most existing methods for community detection find a partition of network nodes, i.e. node communities. In this type of partition, each node is in one and only one community. A link community is a dense subgraph induced by a set of links where there are many links within the subgraph, but few links connecting the subgraph with the rest of the network. Detecting link communities in a partitioning way means to find a partition of network links. In this type of partition, each link is in one and only one community, but a node can belong to multiple communities, depending on the community membership of the links incident on it.

Community detection has many important applications in different fields. For example, in biology community detection has been applied to find protein functional modules [7] and predict protein functions [8]. In sociology, community structure is an important topological feature in considering vaccination interventions of infectious diseases in contact networks [9] and understanding viral propagation in social networks [10].

While most previous studies for community detection have focused on node communities, some recent works have started exploring link communities and cliques [11]–[15]. In some real-world networks, link communities could be more intuitive and informative than node communities, because a link is more likely to have a unique identity while a node often belong to multiple groups [16]–[21]. For example, most individuals in the society have multiple identities such as families, friends, and co-workers, whereas the link between two individuals usually exists for a dominant reason [11]. From the practical point of view, we can naturally detect the overlapping node communities by partitioning the links into communities [13], [16], [22]–[25], because the links connected to a node could belong to different link communities and consequently the node could be assigned to multiple communities of links.

In a recent study [11], the authors define the link density of a link community and the partition density to evaluate the quality of a link community partition. Given a network with 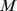 links and 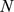 nodes, 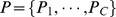 is a partition of the links into 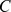 subsets. The number of links in subset 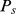 is 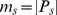. The number of induced nodes is 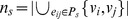. The link density 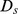 of community 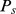 is defined by
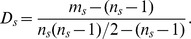



**Figure 1:**
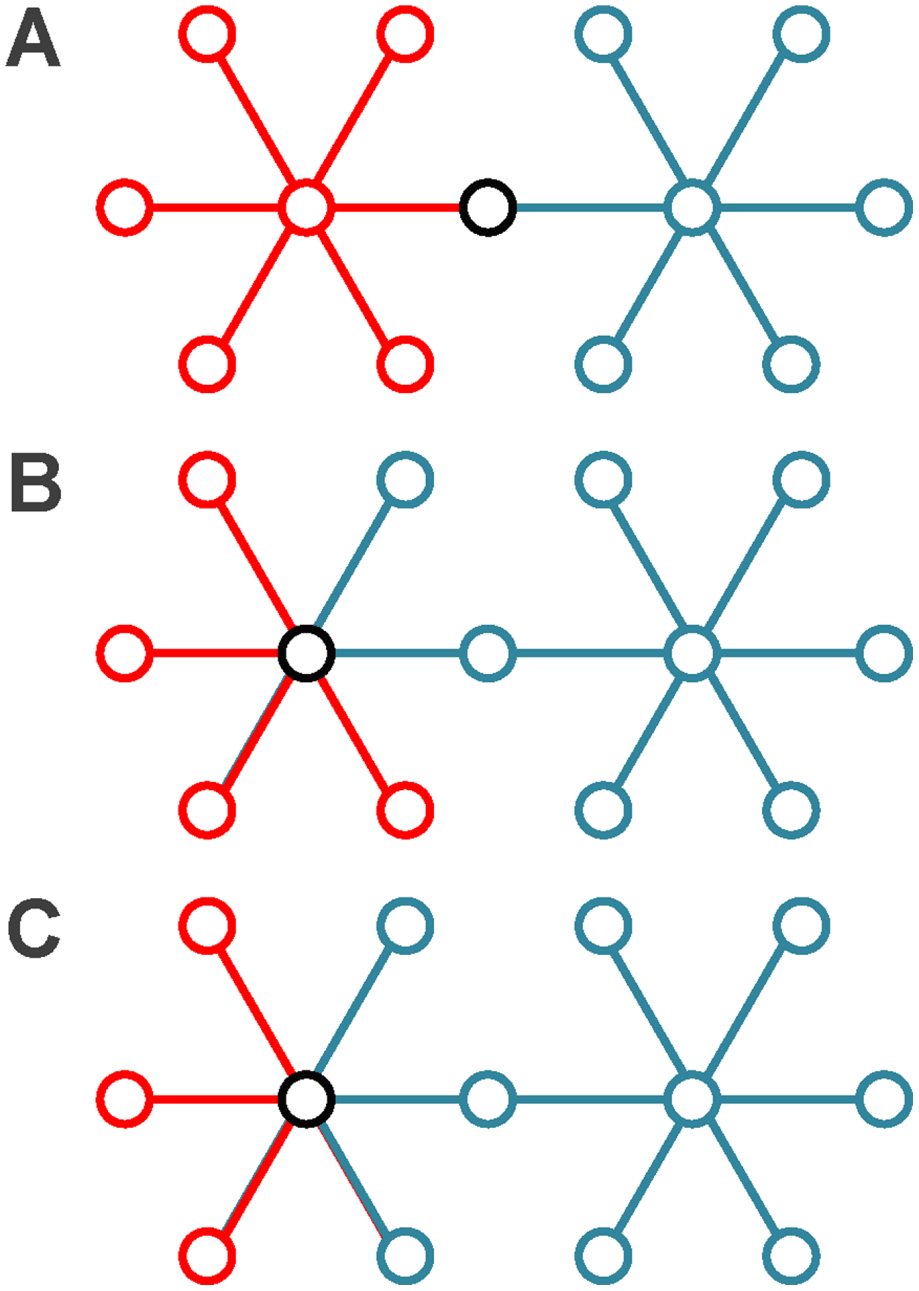
Three different partition results of a tree network. (A) Correct partition. (B,C) Two counter-intuitive partitions. The red links and their adjacent nodes constitute a community, the blue links and their adjacent nodes form another community. The black node is overlapped.

The partition density 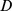 is defined as the average of 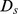, i.e.,
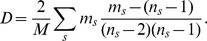



We can see that the maximum value of 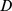 is 1 but it can take values less than 0. 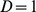 when each community is a clique and 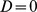 when each community is a tree. When a network is a tree, it cannot be partitioned into proper communities by maximizing 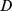, because there are many different optimal partitions, and each partition has the same partition density 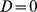. For example, the network in Figure 1 consists of two communities with one overlapping node, and each community is a star graph. If we want to partition the network into two communities by maximizing 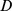, it is difficult to find the correct result shown in Figure 1A, because the partitions in Figure 1B and Figure 1C also have 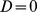.

In most studies on link community partition, each link belongs to one and only one community. But in real-world networks, a link may represent more than one relation between two nodes. For example, two individuals from the same family are also co-workers in the same institute. Consequently two communities may have overlapping links as well. There are few results about how to partition a network into link communities with overlapping links. In this paper, we redefine the partition density of link communities, and formulate the link community partition problem into integer programming models. Then we design a genetic algorithm for solving the link community detection problem and conduct validations on some artificial and real-world networks.

## Methods

### Link Community Partition Density

Given a network with 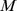 links and 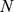 nodes, 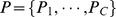 is a partition of the links into 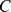 subsets. The number of links in community 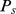 is 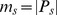. The number of induced nodes from community 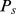 is 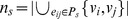. The new link density 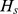 of community 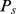 is defined as follows:
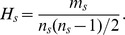



The new partition density 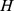 is the average of 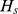:
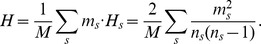



We can see that the maximum value of 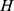 is 1 and the minimum value of 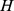 is 0. 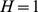 when each community is a clique and 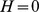 when each community is an empty graph. Given the number of communities, we can find the optimal link community partition by maximizing the value of 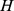. For the network in Figure 1, the partition in Figure 1A has the maximum value of 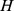, so we can easily find the optimal partition by maximizing 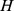.

### Integer Programming Model for Link Community Partition

Given a network 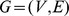 with 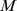 links and 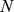 nodes, we assume that the number of link communities is 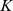 and find the optimal link community partition by maximizing the partition density 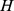. This problem can be formulated into an integer programming model.

Let 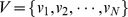 be the node set of 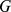, and 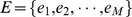 be the edge set of 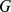. We define 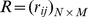 to be the incidence matrix of network 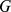, where 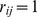 if link 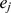 is incident to node 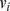, and 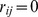 otherwise. We also define binary variables 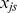 and 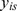 to represent the membership of link 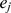 and node 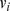 for link community 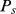:
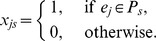


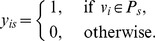



The link community partition problem can be formulated into the following integer programming model–Model-1.
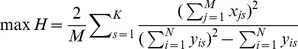
(1)

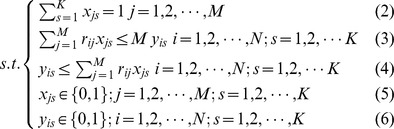



The objective function (1) is to maximize the new link partition density 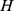. Constraint (2) means that every link belongs to one community. Constraint (3) indicates that if there is one or more links in community 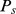 that are incident to node 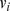, then node 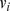 must belong to community 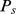. Constraint (4) denotes that if node 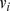 belongs to community 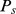, then there is at least one link incident to node 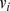 that belongs to community 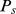.

**Figure 2:**
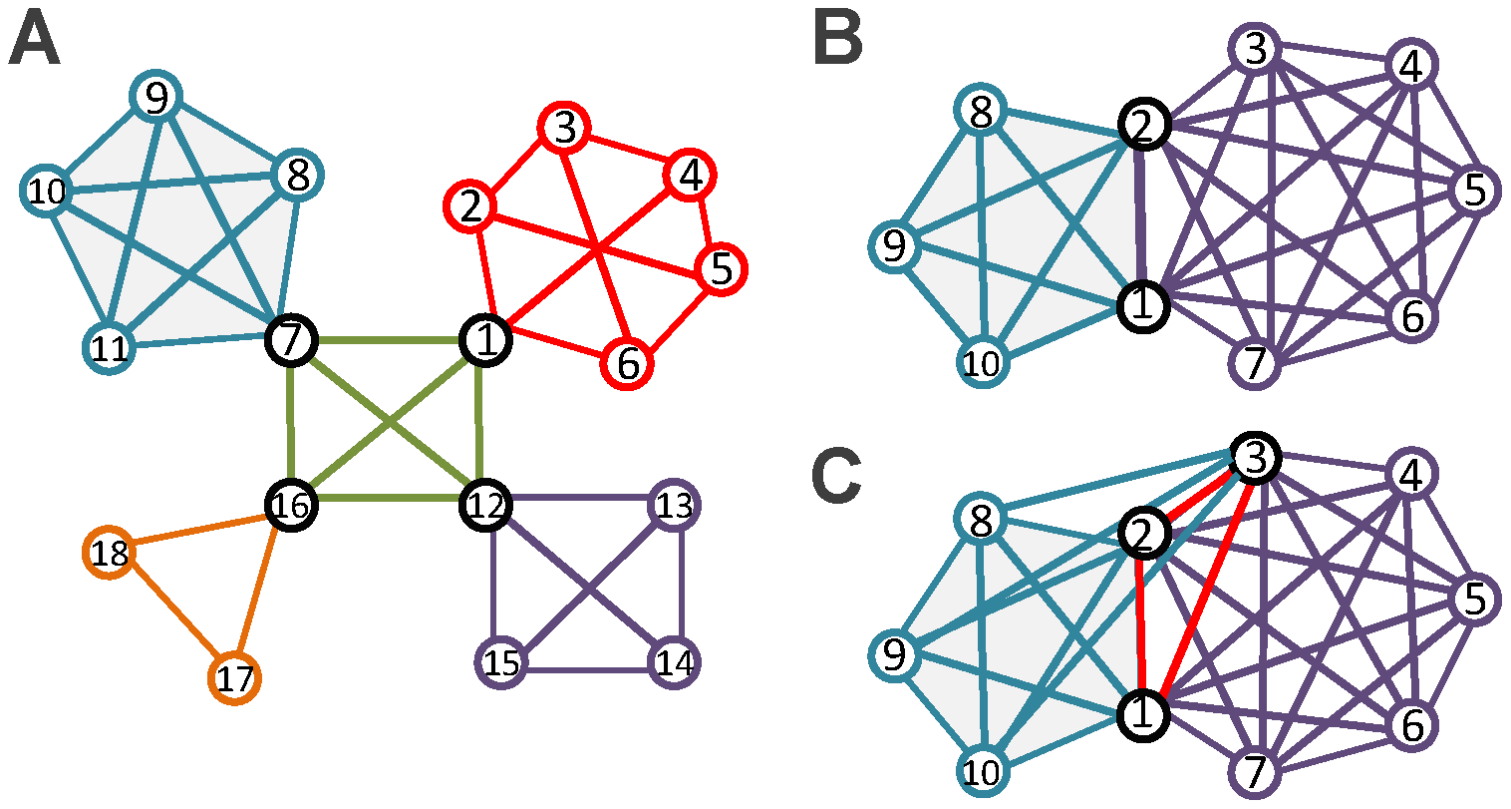
Link communities of three artifical networks. (A) The network consists of five overlapping communities. Nodes 1, 7, 12, 16 are overlapping nodes; (B) The network consists of two overlapping communities. Nodes 1 and 2 are overlapping nodes that belong to the two communities, and link (1, 2) belongs to the two communities as well; (C) The network consists of two overlapping cliques and the overlapped subgraph is a 3-clique.

Since the constraint formulae are simple, we can solve the integer programming model by Lingo software for small networks to see if the model can find overlapping communities properly. Using the quantity function and the integer programming model, we are able to partition several networks into link communities, and obtain correct results. For example, for the network in Figure 2A, we can partition it into five overlapping communities {1, 2, 3, 4, 5}, {7, 8, 9, 10, 11}, {12, 13, 14, 15}, {16, 17, 18}, {1, 7, 12, 16}, and each community is a clique. Nodes 1, 7, 12, 16 are overlapping nodes. The partition density of this link community partition is the optimal objective function value 1. We can partition the network in Figure 2B into two communities with each being a clique. Node 1 and node 2 belong to the two communities and link (1, 2) belongs to the bigger community. The objective function value is less than 1 due to the unique community membership of link (1, 2).

In Model-1, since every link can belong to one and only one community, we might obtain the result that a pair of nodes belongs to the same two communities, but the link between them belong to only one of the communities. For example, in Figure 2B, link (1, 2) only belongs to the bigger community. In fact, node 1 and node 2 may have two different relations. For example, they can be classmates and sisters at the same time. So the link (1, 2) should belong to both classmate community and family community. To address this drawback, we can revise Model-1 and obtain the following model–Model-2.
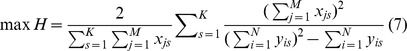


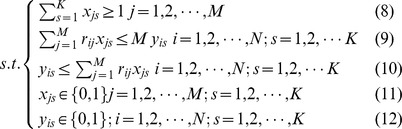



**Figure 3:**
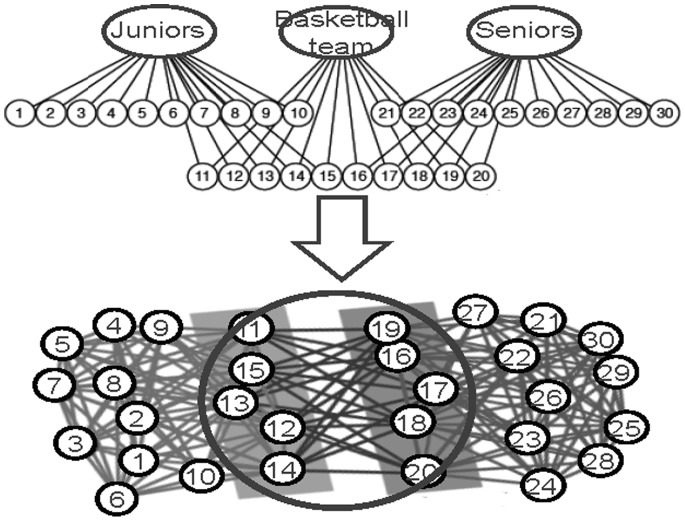
The network in Ref. [11] can be correctly partitioned into three communities by our model, and the objective function value is 1.

In Model-2, the constraint (8) means that every link must belong to at least one community. The link belonging to more than one community is regarded as several links in the objective function (7). Using Model-2, we can partition the network in Figure 2B into the two communities, and link (1, 2) belongs to the two communities as well. Each community is a clique, and the optimal objective function value that the partition corresponds is 1. Figure 2C is a network consisting of two cliques, which are overlapped with a 3-clique. This network can be partitioned into two communities, and each community is a clique. Two overlapping cliques are correctly identified as each link in the overlapping part (3-clique) belongs to the two communities at the same time. The optimal objective function value of the link partition is 1. Figure 3 is an example from reference [11]. In this network, the basketball team community consists of two part members: one part members are from junior community, and the other part members are from senior community. In other words, the basketball team group is completely subsumed in two other groups. Using Model-2, we can partition the network into three overlapping communities and correctly identify the multiple relationships in the basketball team community.

Model-2 can be used to partition sparse networks (e.g., tree-like networks) or even disconnected networks. It is easily to prove that, when a network is disconnected, it can be partitioned into several connected communities. The objective function value is between 0 and 1. Before using Model-2 to partition a network, the number of communities should be given. If the number of communities is unknown, we can use Model-1 to determine it. We can find the maximum partition density for every given number of communities, then compare all the partition densities and find the maximum one. The number of communities with the maximum partition density is the final number of communities.

### Genetic Algorithm for Link Community Detection

Although we can solve Model-2 by Lingo software to partition small-scale networks into link communities, we cannot solve the integer programming model for large-scale networks which is an NP-hard problem. In addition, most of the algorithms for community detection need some *priori* knowledge about the community structure like the number of communities which is impossible to know in real-life networks.

In the following, we will design a genetic algorithm for link community detection. Genetic algorithm (GA) was proposed in [26]. It is a global optimization method in artificial intelligence. When the solution space of a problem is too large to allow exhaustive searching for exact optimal solutions, genetic algorithm can fast converge the problem to a relative smaller solution space, and produces approximately optimal solutions. In [27]–[29], the authors designed genetic algorithms for solving the node community detection problem in unipartite networks or bipartite networks. In this paper, we propose a link community detection algorithm based on the hybrid ideas of genetic algorithm and self-organizing mapping (SOM) algorithm, which aims to find the best link community structure by maximizing the link partition density. The algorithm does not need any *priori* knowledge about the number of communities, which makes the algorithm useful in real-world networks. The algorithm outputs the final link community structure and its corresponding overlapping nodes as the result and does not impose further processing on the output.

#### The GA main functions

First of all, we need to design a chromosome representation encoding the solution for the link community detection problem. In our implementation, the chromosome is represented by a matrix 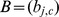, where 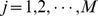, and 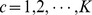. Each element 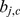 is the strength with which a network link 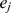 belongs to a community 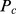. Note that 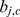 ranges in the interval [0.0, 1.0]. Each link of the network is subject to the following constraint:
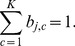
(13)


Equation (13) is to normalize the membership strengths so that the strength sum of a link belonging to all the communities equals 1.

For each chromosome, we design a partition matrix 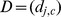, where 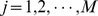, and 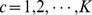. Each element 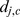 is either 0 or 1. When 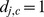, the link 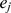 is assigned to community 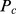, otherwise, link 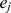 is not assigned to community 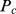. Matrix 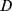 can be calculated from matrix 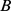 according to the following equation:
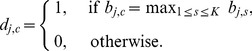
(14)


The network is represented by incidence matrix 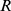, link adjacency matrix 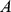 and weighted link adjacency matrix 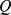. The link adjacency matrix 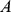 can be calculated by the following equation: 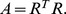 In 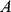, the diagonal elements are 2, and the off-diagonal elements take values in 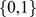 to represent whether two links have a common node or not. Let 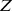 be a diagonal matrix whose diagonal elements are the inverse of nodes’ degree. A node’s degree is the number of links incident to it. In other words,
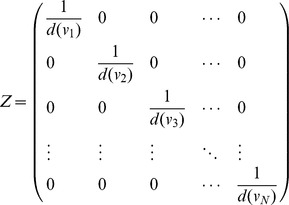



The weighted link adjacency matrix 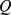 is defined as 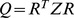, which means the probability for a random walker going from one link to one of its adjacent links across their common node. This can be regarded as the possibility of two adjacent links belonging to the same community.

### The GA Main Functions

- Input

Input the number of nodes 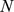 and the number of links 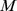 of the network, the maximum number of communities 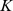. Calculate the incidence matrix 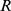, the link adjacency matrix 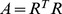, and the weighted link adjacency matrix 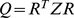. Give the number of individuals 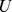, the maximum epoch 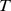, mutation probability 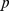, and SOM parameters 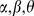.

- Output

Output the link partition matrix 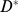 and its fitness value 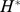 (i.e. link partition density value), the node partition matrix 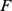. Partition the network into communities according to 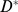 and 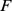.

- Initialization: *t* = 0

Randomly generate an initial population 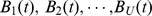, and give an initial values of 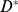 and 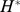.

- Step 1. Population Fitness

For all individuals in the population 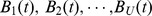, calculate the partition matrices 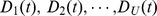, and their fitness values 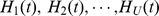.

- Step 2. Population Sorting

Sort 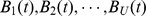 according to their fitness values in descending order. Suppose the sorted chromosomes are 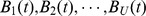, where 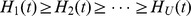. If 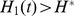, then 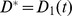, 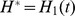. If 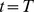, then stop, output 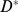 and 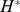, and calculate the corresponding node partition matrix 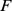. Otherwise, go to Step 3.

- Step 3. Population Crossover

For *i* = 1, …,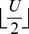, let 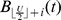 and cross over to produce two temporary individuals (matrices) 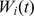 and 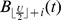. If 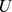 is an odd number, then let 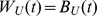.

- Step 4. Population Mutation

Randomly select 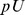 temporary individuals (temporary matrices), and do mutation operation on each temporary individual.

- Step 5. Population SOM

For each temporary individual, do SOM operation on it.

- Step 6. Population Normalization

For each temporary individual, do normalization on it. Denote the normalized individuals by 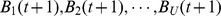. Let 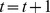, and go to Step 1.

#### Partition matrix and fitness evaluation

For each individual 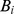, calculate the partition matrix 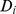 according to the formula (14). For each community 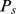, 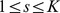, let 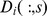 be the 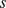-th column of matrix 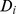. Then 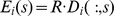 is a column vector whose elements are non-negative integers. A non-zero element in 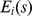 represents that the corresponding node belongs to community 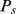. Let 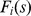 be a 0–1 vector, and 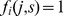 whenever 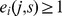. 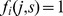 means that node 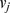 belongs to community 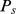. The fitness of individual 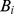 can be calculated by the following equation:
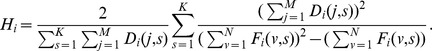



Since there is often one maximum value in each row of matrix 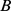, by formula (14), we often partition a link into one and only one community. When a link is an overlapping link of two communities, it cannot be detected by formula (14) directly. To identify the overlapping link correctly, we can replace formula (14) by the following formula (15).
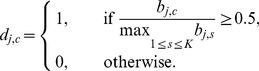
(15)


Using formula (15), an overlapping link can be partitioned into more than one communities.

#### Population sorting

Sort 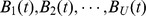 according to their fitness values in descending order. Suppose the sorted chromosomes are 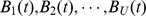, where 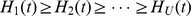. If 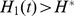, then 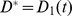, 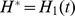.

#### Population crossover

For *i* = 1,2, …,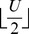, do crossover operation on 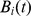 and 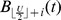 by the following rules: randomly select a column 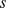, revise the 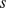-th column of 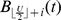 by the 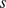-th column of 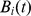, and obtain two new temporary individuals 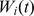 and 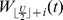. Let 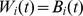. We revise the 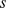-th column of 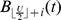 by adding a fraction of the 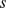-th column of 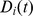 (where 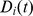 is the partition matrix corresponding to 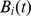), that is,



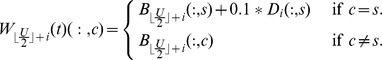



#### Population mutation

According to the mutation probability 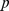, randomly select 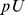 temporary individuals, do mutation operation on each selected individual. For each selected temporary individual 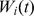, randomly select two parameters 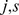, 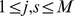. There are three mutation rules that can be used in this genetic algorithm, i.e. exchange the 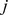-th row and the 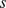-th row in 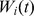, or replace the 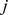-th row by the 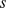-th row in 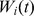, or replace the elements of the 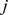-th row with randomly selected numbers in [0.0,1.0]. Three rules lead to insignificant difference in this genetic algorithm. In the following simulation, we replace the 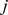-th row with the 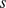-th row in 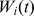. The other elements in 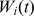 remain unchanged.

#### Population SOM

The Self-Organizing Mapping (SOM) process analyzes the link community ID variance of each link. If the community ID variance of a link is larger than a threshold value, then increase the membership strength of this link for community 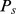 and that of its all neighbor links belonging to the same community. Meanwhile, decrease the membership strengths of all non-neighbor links for community 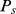. If the community ID variance of a link is smaller than the threshold value, the membership strength of the link and all neighbor links belonging to the same community decreases. This process can improve the quality of the partition by eliminating wrongly placed links due to the behaviors of the algorithm.

For 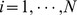, do SOM operations on individual (chromosome) 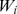 as follows:

- Calculate its partition matrix 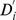 from the matrix 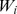 according to the formula (14);
- For 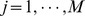, do the following operation on link 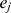.
- Find the community ID of link 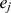 which corresponds to the maximum element in the 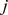-th row of 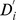 (the maximum element must be 1). Suppose the maximum element in the 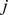-th row of 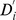 is in the 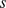-th column, which is 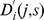. This means that link 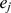 belongs to community 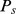.
- Calculate the total number 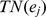 of adjacent links of 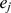 (including edge 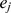), and the number of adjacent links in 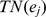 belonging to community 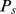 (denoted by 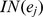). 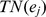 is equal to the sum of elements in the 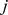-th row of matrix 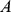, which can be expressed by 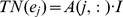, where 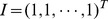, and 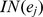 can be obtained by the equation 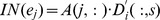.
- Calculate the community ID variance 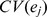 of link 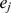 by the following equation.

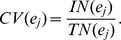

- If 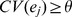, then

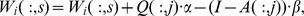



otherwise,
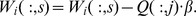
where 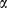 and 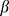 are adjustable parameters that decrease with the step 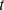 (In this paper, we let 
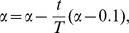
 
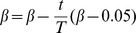
). In the above equations, if an element is negative, then we set it to be 0.01.

#### Normalization

Since the sum of row elements in temporary matrix 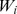 might not be 1, we should do normalization on each row of matrix 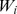. For 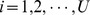, do normalization on each row of temporary matrix 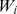 through dividing it by the sum of row elements.

#### Complexity of the genetic algorithm

The running time of the genetic algorithm is mainly determined by the running time of Step 1 and Step 5. The complexity of Step 1 is at most 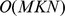, and the complexity of Step 5 is at most 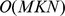. So the complexity of the genetic algorithm is 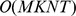.

## Results

In this section, we apply the genetic algorithm to a class of artificial networks and several real-world networks, and analyze the results in terms of classification accuracy and ability of detecting meaningful communities. The algorithm is implemented by Matlab version 7.1.

We first do validations on the networks described in Figure 2. By setting the parameters as described in Table 1, we can find all the optimal partitions. Then we conduct validation experiments on several types of overlapping networks with special structures and several real-world networks.

**Table 1:** The parameters used in the GA algorithm for solving the link community detection problem on networks in Figure 2.

network	*K*	*N*	*p*	*θ*	*α*	*β*	*T*
A	5	40	0.3	0.2	1.0	0.2	2000
B	2	40	0.3	0.3	1.0	0.2	600
C	2,3,4,5	40	0.3	0.2	1.4	0.1	600

### Ring Networks Consisting of Cliques

**Figure 4:**
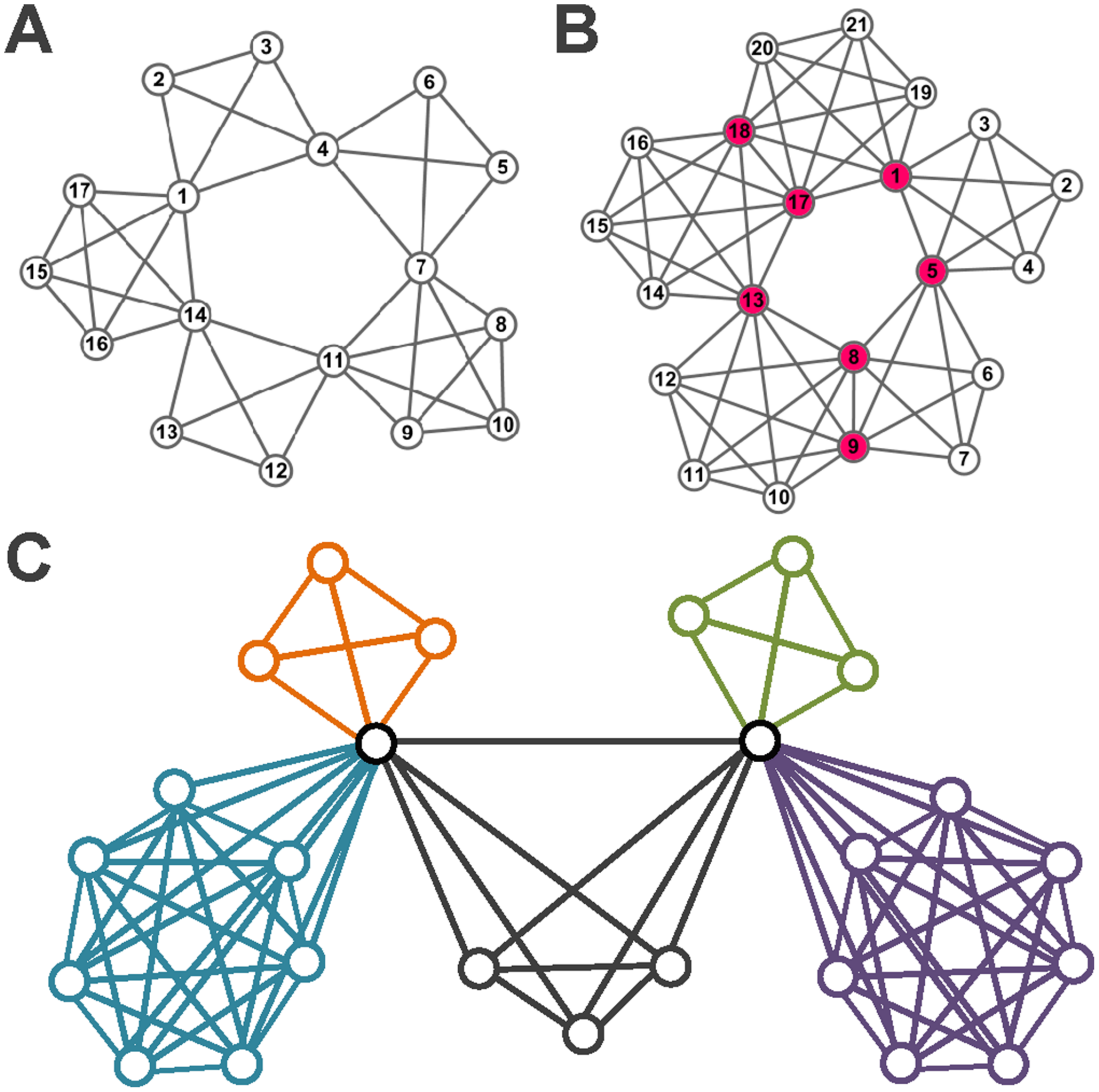
Link communities of three networks of heterogeneous cliques. (A) The ring network of heterogeneous cliques. Each community is a clique, and two adjacent communities are connected by one node. (B) The ring network of overlapping heterogeneous cliques. Each community is a clique, and two adjacent communities are connected by one node or one link. (C) The tree network of heterogeneous cliques. Each community is a clique, and two adjacent communities are overlapped by one node [11].

We test our algorithm on a type of exemplar networks, that is, rings of cliques, which is not the same as in [30]–[32]. This network consists of many heterogeneous cliques, connected through single nodes (Figure 4A). Each clique 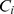 (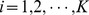) is a complete graph. The network has a clear link modular structure where each community corresponds to a single clique, thus the optimal partition density is 1. Using our genetic algorithm, we can easily detect the optimal partition and identify the overlapping nodes. Figure 4A demonstrates a network consisting of two 4-cliques and three 5-cliques. Our method can obtain the optimal partition and identify the overlapping nodes correctly.

We also test our algorithm on an overlapping ring network of cliques. The network consists of many heterogeneous cliques, and two adjacent cliques are overlapped by several nodes and links (these overlapping nodes and links form a small clique) (Figure 4B). The overlapping ring of clique network can be partitioned into multiple communities by our genetic algorithm, and each community is a clique. The overlapping small cliques connecting pairs of large cliques can also be correctly identified.

We further validate our algorithm on a tree network of cliques. This network consists of multiple cliques connected by overlapping nodes. Many subnetworks of metabolic networks are similar to a tree of cliques. The network we test consists of five cliques depicted in Figure 4C. Using our genetic algorithm, the network can be partitioned into the five cliques, and the fitness (partition density) of the partition is 1.

### Applications on Real-world Networks

In this subsection, we validate our method on three real-world networks.

#### The karate club network

**Figure 5:**
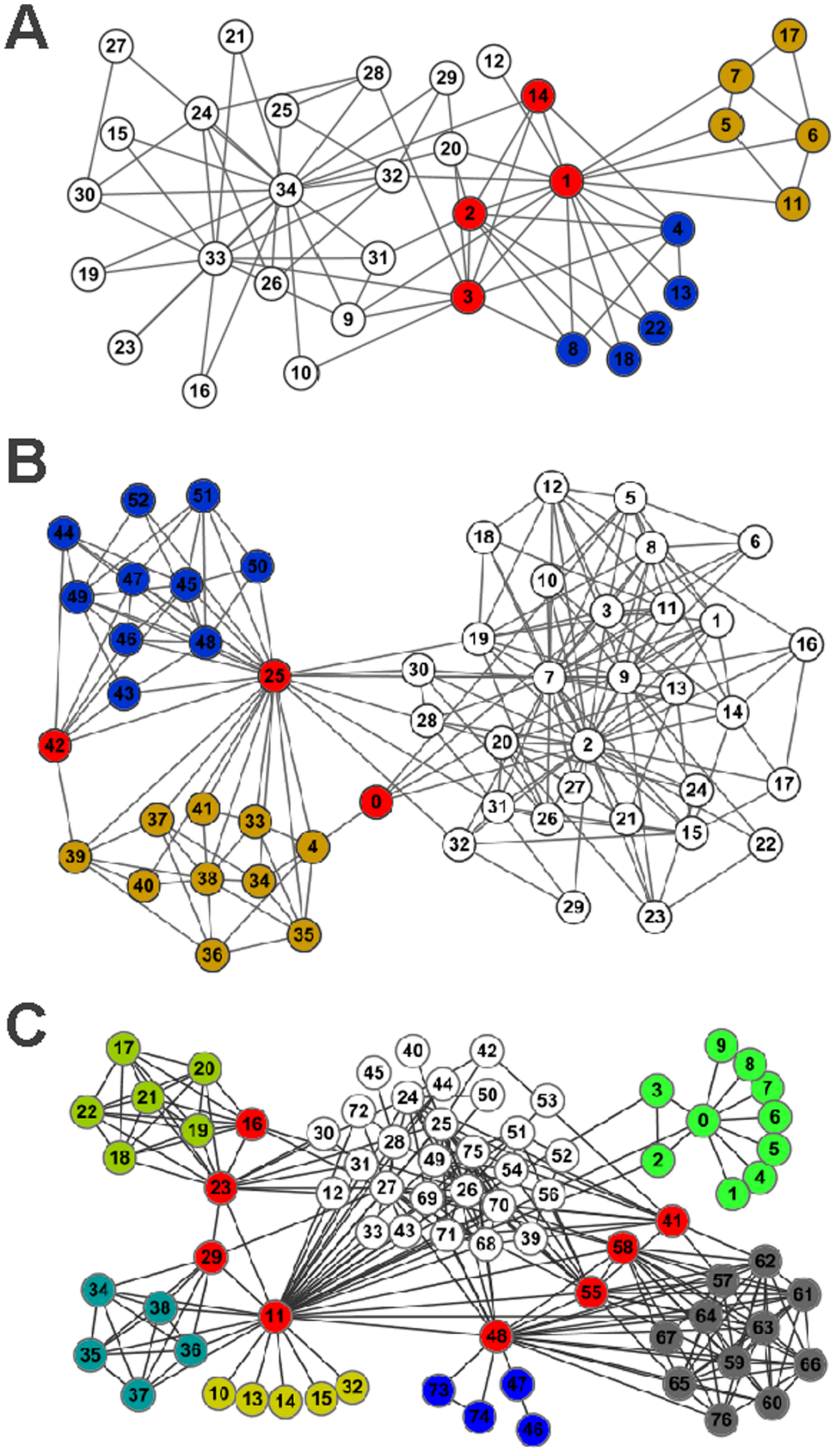
Link communities of some real-world networks. (A) The Karate club network; (B) The word association network; (C) The co-appearance network.

The first example we consider is the famous karate club network analyzed by Zachary [33]. It has also been analyzed by many community detection studies. It consists of 34 members of a karate club as nodes and 78 edges representing friendship between members of the club which was observed over a period of two years. We apply our method to the karate club network using the parameters 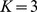, 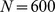, 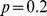, 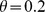, 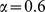, 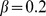, 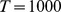. The result is illustrated in Figure 5A. The average link density is 0.3349. The colors of the links indicate the link communities detected by our genetic algorithm, and the colors of the nodes indicate the node communities deduced from link communities. In this karate club network, our link communities show that node 1 belongs to three communities, and nodes 2 and 3 belong to two communities. The overlapping part is a 3-clique which was not identified by previous methods.

#### Word association network

The word association network is picked from the South Florida Free Association norm list (http://www.usf.edu/FreeAssociation/). In the South Florida Free Association norm list, the weight of a directed link from one word to another indicates the frequency with which the people in the survey associate the end point of the link with its starting point. The word “play” association network has been replaced with an undirected one and tested in [34]–[36]. This network has 53 nodes representing different words and 197 association edges. Using the genetic algorithm with parameters 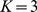, 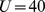, 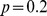, 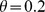, 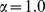, 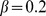, 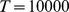, we can partition this network into three overlapping communities with the fitness (objective function) value 0.3396. The result is described in Figure 5B. From the partition results, we can see that words with frequent associations are in the same communities. In this network, the word “play” is strongly associated with most words, so it is an overlapping node. This result has also been obtained by a graph-theoretical method for node community detection [35].

#### The co-appearance network

The co-appearance network contains 77 characters in the novel Les Mis*é*rables by Victor Hugo. There are 77 nodes and 254 links in the co-appearance network. The nodes represent 77 characters and the links connect any pair of characters that appear in the same chapter of the book. This network was compiled by Knuth [37] based on the list of characters’ appearance by scene. In this paper, we use the unweighted network. Figure 5C shows the partition obtained by our genetic algorithm, which divides the network into seven overlapping communities. The resulting partition agrees reasonably well with the social divisions and subplots in the plot-line of the novel. In [16], the network is partitioned into five communities.

From the results, we can see that this network contains some highly connected nodes, some of which (nodes 11, 16, 23, 29, 41, 48, 55, 58) are overlapping nodes and can connect to multiple communities of the network. These nodes can cause serious problems if we want to partition the network by conventional node community schemes because they do not fit adequately to any community. No matter which community we place a highly connected node in, its outside links are more than its inside links. In contrast, link community schemes can provide an elegant solution to this problem because they allow a node to belong to multiple communities. As shown in Figure 5C, our algorithm properly places nodes 11, 16, 23, 29, 41, 48, 55, 58 into more than one community. These nodes correspond to the major characters in the novel. In addition, our algorithm also classifies the major characters of the novel into their proper communities. For example, node 48 corresponds to Gavroche, who is assigned to three communities, corresponding to his family members, friends, and the people with battle respectively.

## Discussion and Conclusion

Community structure is one of the main characteristics of complex networks and detecting community structure is very helpful for understanding the functions of these networks. In this paper, we investigate the link community detection problem and propose a new quantity function for link community detection. We formulate the link community identification problem into an integer nonlinear programming model based on the proposed quantity function. Furthermore, we design a GA algorithm for solving the link community detection problem and conduct validation experiments on some artificial and real-world networks.

The extensive computational results demonstrate that our model and algorithm can detect overlapping communities effectively. It will be promising to apply and test our method onto real large-scale networks. Generally, note that the real large-scale networks are very sparse. According to the computational complexity analyzed before, it will be feasible to apply it onto sparse networks with about 10000 nodes. This method can be easily extended to detect the communities of both directed networks and bipartite networks, which will be further explored in our future study.
